# “Separated” precise subsegmentectomy: Single‐port thoracoscopic noncombined subsegmentectomy in one lung lobe

**DOI:** 10.1111/1759-7714.14746

**Published:** 2022-11-24

**Authors:** Shuliang Zhang, Maohui Chen, Yizhou Huang, Guanglei Huang, Taidui Zeng, Wei Zheng, Chun Chen, Bin Zheng

**Affiliations:** ^1^ Key Laboratory of Cardio‐thoracic Surgery (Fujian Medical University) Fujian Province University; ^2^ Department of Thoracic Surgery Fujian Medical University Union Hospital Fuzhou China

**Keywords:** combined dimensionality reduction method, video‐assisted thoracoscopy, noncombined subsegmentectomy, three‐dimensional reconstruction and simulation

## Abstract

**Background:**

In clinical practice, combined segmental resection (CSS) can avoid resection of multiple segments to preserve lung function. When two or more distant lung segments or subsegments of the same lobe present with a ground glass opacity (GGO) that meets the indications for sublobar resection, conventional CSS or wedge resection could not remove all the nodules, and lobectomy is performed in most of these patients. For these particular types of nodules, we perform a single lobe noncombined subsegmental resection, or “separated” precise subsegmentectomy, to preserve more lung tissue. This study was designed to initially assess the feasibility and safety of “separated” precise subsegmentectomy.

**Methods:**

Selected cases of specific GGO were subjected to “separated” precise subsegmentectomy and the results of general clinical data, perioperative operative time, bleeding, length of stay, computed tomography (CT) review, lung function and its dynamic changes were collected and analyzed in these patients.

**Results:**

“Separated” precise subsegmentectomy was performed in 12 patients and successfully completed. The median operation time, bleeding amount, and length of hospital stay were 96 min, 50 ml and 4 days, respectively. There was one case of pulmonary infection and one case of persistent air leakage, no death or pulmonary torsion, bronchopleural fistula and other pulmonary complications occurred. After 3 months, the median percentage of lung function retention was 91.7%, and the CT scan showed that the reserved lung tissue of 12 patients was well inflated and there was no obvious imaging manifestation of atelectasis.

**Conclusion:**

“Separated” precise subsegmentectomy is a novel and safe surgical method that provides a more optimized way for patients with specific multiple nodules to preserve lung function. Further prospective large studies are needed to verify this finding.

## Graphical abstract

In clinical practice, when two or more distant lung segments or subsegments of the same lobe present with a ground glass opacity, lobectomy is performed in most of these patients. For these particular types of nodules, we perform “separated” precise subsegmentectomy to preserve more lung tissue. “Separated” precise subsegmentectomy is a novel and safe surgical method that could provide a more optimized way for patients with specific multiple nodules to preserve lung function.

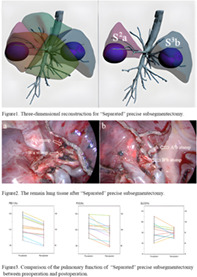

## INTRODUCTION

In 1995, a prospective randomized study conducted by the North American Lung Cancer Research Group (LCSG) concluded that lobectomy was superior to sublobectomy in terms of recurrence rate and prognosis.^1^ Sublobar lobectomy is commonly used as a surgical method for patients with poor lung function and advanced age.^2^ However, recent studies have shown that segmentectomy can achieve the same results as lobectomy for ground glass opacity (GGO) that meets the indications for segmental lung surgery, and the safety and efficacy of segmental lung resection is gradually being proven.^3^, ^4^, ^5^ The JCOG0802 study showed that 5‐year survival for lung segmental resection for peripheral lung cancer with consolidation tumor ratio (CTR) >0.5 and a total diameter ≤2 cm was comparable to lobectomy.^6^ For centrally located ground glass lung nodules, segmental lung resection allows for more normal lung tissue to be preserved with less loss of lung function than lobectomy.^7^, ^8^ However, if there are two or more pulmonary nodules in one lobe at the same time, it could be difficult to make clinical decisions. If multiple nodules are located at the hilum or scattered across multiple lung segments, lobectomy is the optimal approach. In addition, combined segmental resection (CSS) is recommended for nodules located between lung segments or multiple nodules located in adjacent lung segments. For patients with small peripheral c‐T1N0M0 NSCLC involving multiple segments, CSS only removes the subsegments involved in the tumor. Compared with the resection of the whole segment involved in the tumor, CSS can save more subsegments, so that the forced expiratory volume in 1 second (FEV1) retained by each lung lobe and the value retained by each subsegment are higher than in multisegmental pneumonectomy.^9^ However, for multiple nodules located in nonadjacent lung segments or subsegments, CSS is difficult to remove all pulmonary nodules at the same time, lobectomy may be considered to remove all multiple pulmonary nodules, but at the expense of the whole lung lobe.

In this study, noncombined subsegmental resection of specifically located multiple pulmonary nodules was performed under the guidance of three‐dimensional reconstruction. In this previously unreported method, subsegmental resection of two pulmonary nodules in the same lobe was performed. The subsegments involved were relatively independent at the same lung lobe of resection to retain more lung tissue on the basis of resection indications. The surgical method was named “separated” precise subsegmentectomy. The feasibility and safety of this method were preliminarily evaluated by observing the short‐term perioperative results and postoperative imaging findings.

## METHOD

### Patient selection

Selected patient data were retrospectively collected between January 2021 and December 2021. The eligibility criteria were (1) age 18–75 years, (2) multiple nodules in a single lobe with a major lesion or all lesions meeting the indications for segmental lung resection, i.e. CTR <0.5 and total diameter ≤2 cm, and (3) undergoing single lobe noncombined two‐pulmonary segmentectomy or subsegmentectomy. Routine preoperative examinations included chest thin‐slice computed tomography (CT), brain magnetic resonance imaging (MRI), echocardiography, electrocardiogram, color Doppler ultrasound of supraclavicular lymph nodes and whole abdomen, pulmonary function tests, and whole‐body bone scan. The preoperative and postoperative staging were performed according to the 8^th^ edition of the TNM Classification for Lung Cancer. All intraoperative and postoperative complications and clinical examination data were also recorded.

This study was approved by the Ethics Committee of Fujian Medical University Union Hospital, and the patients were informed about the operative methods and signed the informed consent before operation. All operations were performed by experienced chief physicians with professional expertise in pulmonary resection.

### Preoperative three‐dimensional reconstruction

**FIGURE 1:**
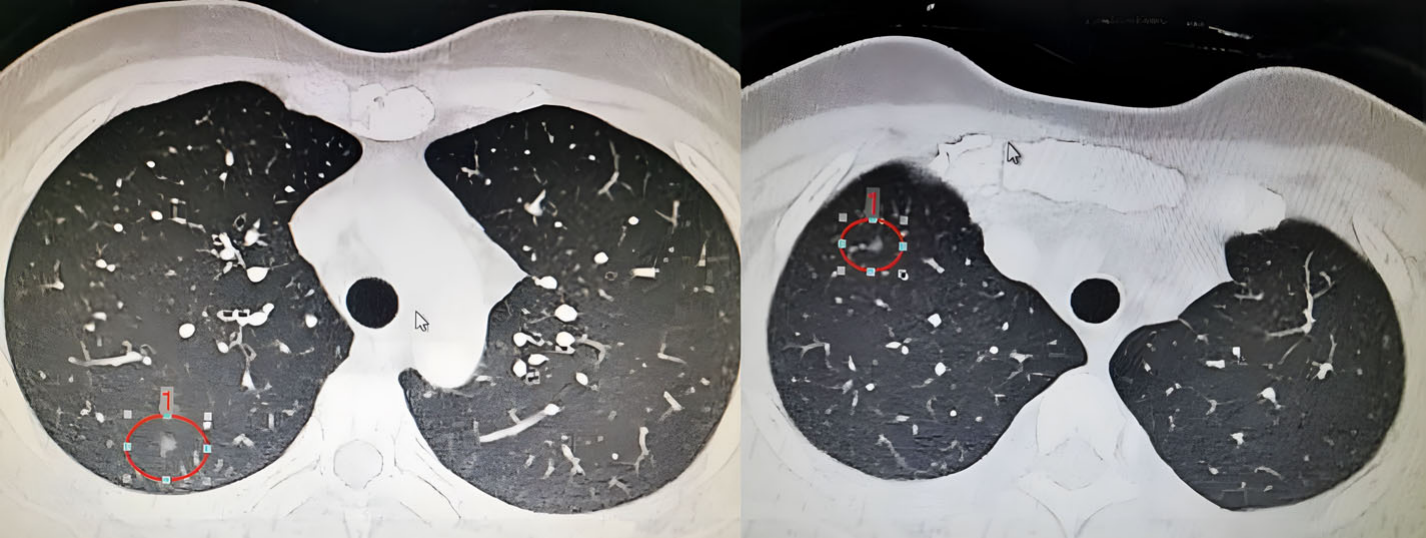
(a) Preoperative lung CT scan for pulmonary node in RS2a; (b) Preoperative lung CT scan for pulmonary node in RS3b

**FIGURE 2:**
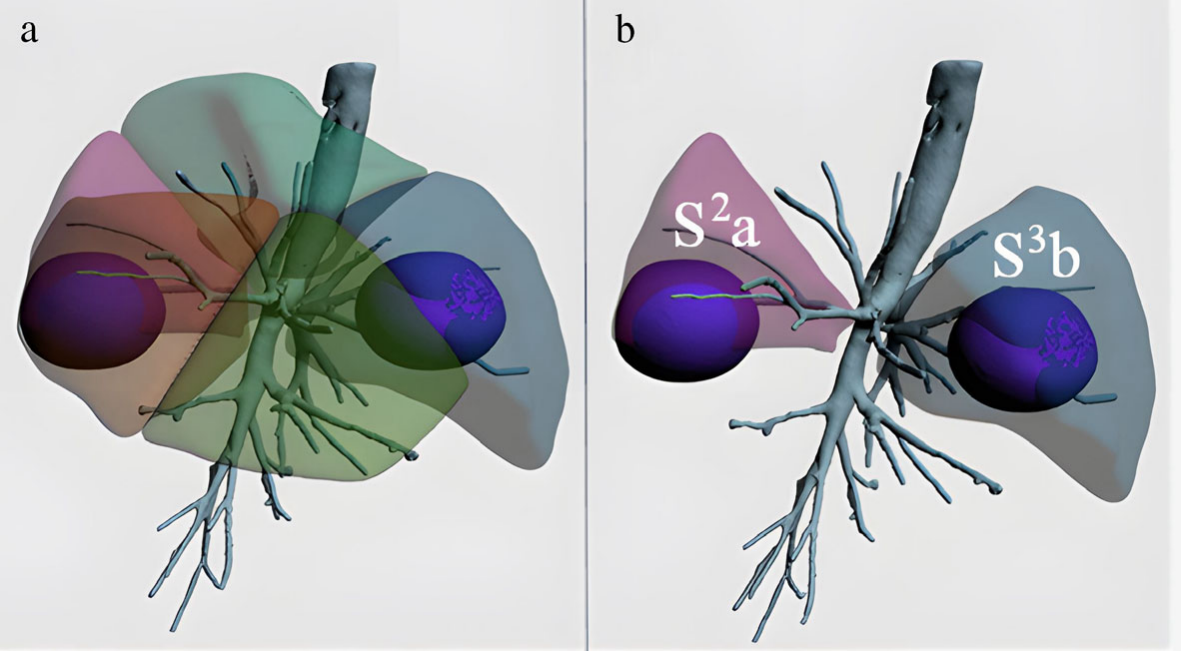
(a) Three‐dimensional reconstruction for noncombined subsegmentectomy of S2a + S3b. The blue spheres in the subsegments indicate the safe resection margins. (b) “Separated” precise subsegmentectomy planned to be resected after the surgeon evaluates the resection plan

Preoperative three‐dimensional reconstruction was performed with thin‐slice enhanced CT (Figure 1) as data source using the IQQA‐3D system (IQQA‐3 D; developed by IQQA‐Chest, EDDA Technology). The pulmonary regions were planned and reconstructed accurately according to the trachea, arteries, and veins of the tracheal branch and the lung lobe to clarify the positional relationship between the lesion and the segmental structure. The location and extent of pulmonary nodules were marked and the resection marginal pellets were made 2 cm away from the edge of the lesion. The sufficient margin is defined as margin ≥2 cm or the ratio (margin/tumor diameter) ≥1. The adequacy of the margin was evaluated according to the simulated resection range, and additional resection was performed on the adjacent segments or subsegments if the margin was not enough. All the cases reported in this study underwent noncombined subsegmental resection, the sufficient margin was included in the target subsegment, and virtual surgery was performed to verify the surgical scheme of noncombined subsegmental resection (Figure 2).

### Operation processes

After general anesthesia of the patient, surgery was performed with single‐hole thoracoscopy. An incision of 3.5–4.0 cm was made at the fourth intercostal space at the midaxillary line. The target arteries and bronchi were separated and exposed along the upper hilum, then transected with an ultrasonic knife after ligation. Both lungs were inflated with 100% oxygen and one‐lung ventilation for 15 min depressed the target lung tissue.

**FIGURE 3:**
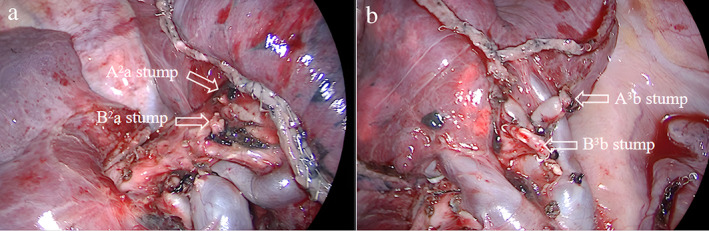
(a) The remain lung tissue after S2a resection. (b) The remaining lung tissue after S3b resection

Our research team uses the joint dimensionality reduction method to obtain and deal with the intersegmental plane. For the treatment of the intersegmental plane, according to the guidance of the intersegmental expansion‐collapse boundary, the intersegmental parenchyma is separated distally from the hilum with an ultrasonic knife, the bronchial stump is lifted away from the hilum, the target pulmonary segment is stretched to one side, and nearly two‐thirds of the proximal parenchyma is carefully separated, so that the remaining target parenchyma is thin enough and located on a two‐dimensional plane. At this point, the stapler can be quickly positioned in the resection plane to cut the remaining substance (Figure 3).

The lung leakage test was performed after mediastinal lymph node sampling. The bronchial stump was checked for obvious air leakage and bleeding, and hemostatic materials were placed on the surgical wound. If a small amount of air leakage was found, it was treated with fibrin sealant spray (Shanghai Race) and covered with absorbable polyglycolic acid reinforced felt (NEOVEIL). When no bronchopleural fistula was found during the operation, two drainage tubes (24‐28F drainage tube and 8‐F ultra‐fine drainage tube) were inserted into the thoracic cavity. The postoperative drainage volume was the sum of closed drainage tube and ultra‐fine drainage tube. The time to remove the closed drainage tube should meet the following requirements: (i) no lung air leakage, (ii) good lung reinflation, and (iii) the ultra‐fine drainage tube should be discharged smoothly.The ultra‐fine drainage tube would be removed if the liquid output was less than 150 ml for 24 h.

### Statistical method

SPSS 22.0 software was adopted for statistical analysis. The data that did not meet a normal distribution or conformed to a skewed distribution were expressed as the median and interquartile range [M(P25–P75)].

### Follow‐up action

Pulmonary CT (Figure 3) and pulmonary function were examined again 3 months after operation. The percentage of postoperative pulmonary function retention (PPF%) was calculated using the following formula: (postoperative FEV1/preoperative FEV1) × 100 (%).

## RESULTS

Twelve patients from the same treatment group underwent single‐lobe noncombined subsegment surgery in one center. The operation was performed successfully in all 12 patients, including five males and seven females, with an average age of 48 years (range 33–65 years). Ten patients underwent single‐hole thoracoscopic resection of the lesions and two patients underwent Davinci robot thoracoscopic resection of the lesions. Postoperative pathology showed that 10 patients had microinvasive adenocarcinoma, one patient had invasive adenocarcinoma, two patients had adenocarcinoma in situ, and no metastatic carcinoma was found in the lymph nodes (Table 1).

**TABLE 1:** Characteristics of patients receiving “separated” precise subsegmentectomy

Patient	Age	Sex	Position	Tumor size (mm)	Area of resection	CTR	Preoperative follow‐up time (months)
1	60	Female	RUL	6.5/6.8	RS2a + S3b	0.25/0.00	12
2	33	Male	RUL	8.1/5.6	RS1b + S2a	0.37/0.00	4
3	50	Female	LUL	10.0/4.8	LS1 + 2c + S3c	0.43/0.00	35
4	50	Female	RUL	4.5/5.3	RS2a + 3b	0.00/0.00	42
5	65	Female	RUL	5.0/6.7	RS1b + S2a	0.00/0.00	17
6	58	Male	RUL	13.0/5.1	Rs2a + s3b	0.45/0.00	6
7	32	Female	RLL	11.0/8.3	RS6a + S8a	0.48/0.25	6
8	42	Female	LUL	6.0/5.2	LS1 + 2a + S4a	0.27/0.00	50
9	43	Male	RLL	11.0/4.9	RS6b + S8b	0.50/0.00	6
10	49	Female	RLL	5.0/6.7	RS6a + S8a	0.00/0.00	4
11	45	Male	LUL	9.0/6.4	LS1 + 2b + S4a	0.39/0.00	10
12	49	Male	LUL	6.0/5.3	LS1 + 2a + S5a	0.23/0.00	3

Abbreviations: CTR, consolidation tumor ratio; LUL, left upper lobe; RLL, right lower lobe; RUL, right upper lobe.

The data were collected and analyzed as follows (Table 2): median tumor diameter 6.93 mm (range 4.5–13.0), median operation time 103.67 min (range 88.0–139.0), median bleeding amount 49.58 ml (range 20.0–100.0), postoperative median extubation time of closed drainage tube 2.42 days (range 1.0–5.0), median postoperative drainage amount 466.83 ml (range 230.0–640.0), and postoperative median length of hospital stay 3.83 days (range 2.0–6.0). All patients were successfully discharged from hospital, and there were no death cases. One patient had pulmonary infection and one patient had pulmonary torsion. There were no other pulmonary complications such as pulmonary infection, pulmonary torsion, bronchopleural fistula (Table 3).

**TABLE 2:** Surgical data of patients receiving “separated” precise subsegmentectomy

Patient	Operative time (min)	Blood loss (ml)	Air leak (during operation)	Surgical incision (single port)	Anesthesia method	Pathology results (intraoperative frozen section)	Lymphatic metastasis	Inflation‐deflation method
1	90	35	No	Yes	GA	MIA/MIA	No	Yes
2	129	50	No	Yes	GA	MIA/MIA	No	Yes
3	98	50	Yes	Yes	GA	MIA/AIS	No	Yes
4	88	100	No	Yes	GA	AIS/MIA	No	Yes
5	96	30	No	Yes	GA	MIA/MIA	No	Yes
6	93	50	Yes	Yes	GA	IAC/MIA	No	Yes
7	127	20	No	Yes	GA	MIA/MIA	No	Yes
8	105	50	No	Yes	GA	MIA/MIA	No	Yes
9	139	100	Yes	Yes	GA	MIA/MIA	No	Yes
10	90	50	No	Yes	GA	MIA/MIA	No	Yes
11	93	30	Yes	Yes	GA	MIA/MIA	No	Yes
12	96	30	No	Yes	GA	MIA/MIA	No	Yes

Abbreviations: AIS, adenocariconoma in situ; GA, general anesthesia; IAC, invasive adenocariconoma; MIA, microinvasive adenocarcinoma.

**TABLE 3:** Postoperative data of patients receiving “separated” precise subsegmentectomy

Patient	Complications	Extubation time (days)	Pulmonary torsion	Pleural effusion (ml)	Postoperative hospital stay (days)	Pathology results	Follow‐up time after surgery (months)	PPF
1	No	2	No	330	3	MIA/MIA	17	97.0%
2	No	2	No	230	3	MIA/MIA	12	96.6%
3	Pulmonary infection	3	No	440	4	MIA/AIS	20	90.5%
4	No	2	No	425	4	AIS/MIA	18	91.7%
5	No	2	No	477	4	MIA/MIA	22	98.0%
6	Pulmonary torsion	5	Yes	340	6	IAC/MIA	12	87.8%
7	No	2	No	535	4	MIA/MIA	21	96.0%
8	No	2	No	555	3	MIA/MIA	15	90.6%
9	No	3	No	555	5	MIA/MIA	13	97.6%
10	No	1	No	575	2	MIA/MIA	16	91.5%
11	No	3	No	500	4	MIA/MIA	17	91.4%
12	No	2	No	640	4	MIA/MIA	21	91.6%

Abbreviations: AIS, adenocariconoma in situ; IAC, invasive adenocariconoma; MIA, microinvasive adenocarcinoma; PPF, preserved pulmonary function.

**FIGURE 4:**
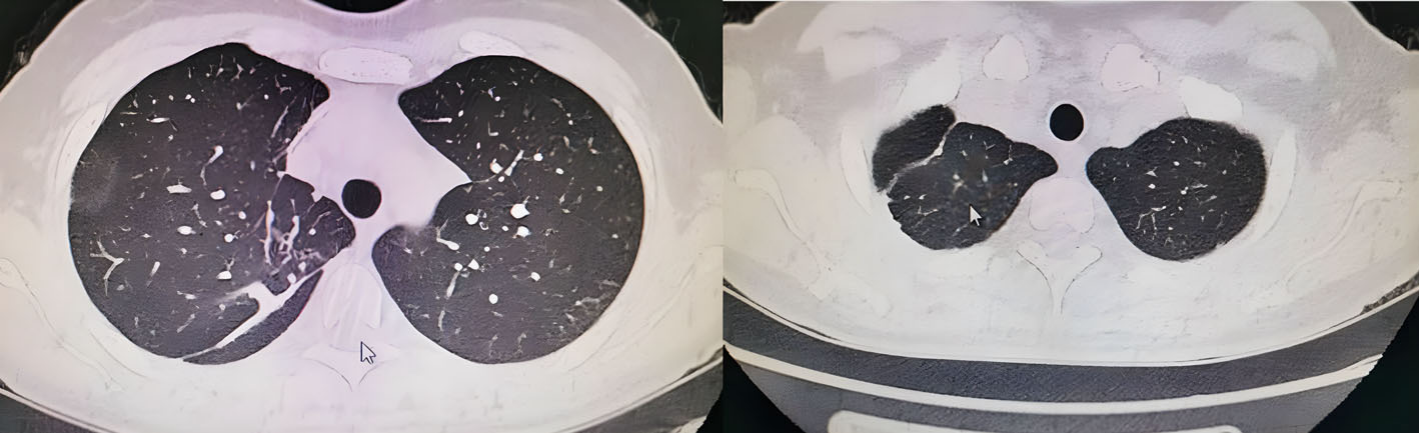
(a) (b) The postoperative lung CT scan for RS3b + S2a after 3 months.

**FIGURE 5:**
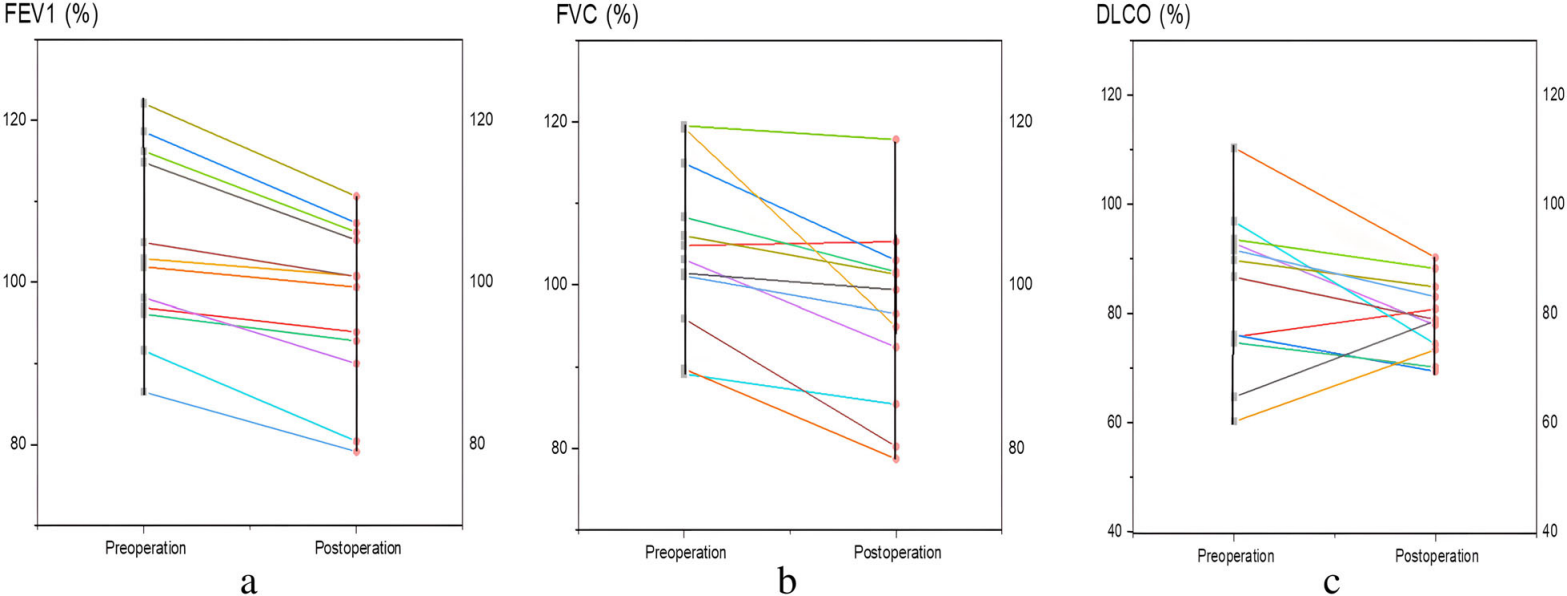
(a) Comparison of the FVC% of “separated” precise subsegmentectomy between preoperation and postoperation. (b) Comparison of the FEV1% of “separated” precise subsegmentectomy between preoperation and postoperation. (c) Comparison of the DLCO% of “separated” precise subsegmentectomy between preoperation and postoperation. FEV1, forced expiratory volume in 1 s; FVC, functional vital capacity; DLCO, diffusion capacity of carbon monoxide

During the follow‐up period, there was no pleural effusion, second admission or second operation. The CT scan was reviewed 3 months after surgery, and the imaging findings showed that the preserved lung tissue was well inflated with no obvious atelectasis (Figure 4). Lung function was examined at 3 months after surgery, with a median PPF% of 91.7% (range 87.8–98.0%). Figure 5. shows the comparison of FVC%, FEV1%, and DLCO% in preoperative and postoperative noncombined subsegmental resection. Compared with preoperative pulmonary function, postoperative pulmonary function showed an overall downward trend, but the downward trend is acceptable.

## DISCUSSION

With the development of imaging technology and the improvement of people's awareness of physical examination in China, the detection rate of multiple pulmonary nodules is increasing, especially multiple GGO. When lung function permits, one‐stage resection of multiple lung ground glass shadows with indications for resection can be considered. It is reported that the prognosis of patients with multiple GGO after surgical resection is satisfactory and even segmental pneumonectomy does not affect the prognosis.^10^ Lobectomy and systematic lymph node dissection have been standard procedures for early non‐small cell lung cancer for a long time.^11^ However, most early studies that showed the superiority of lobectomy was not properly randomly grouped, did not distinguish between wedge resection and segmental pneumonectomy, and did not consider the factors affecting survival, such as tumor size and the type of lymph node dissection. In addition, the follow‐up of patients was often incomplete, which led to misleading conclusions in favor of lobectomy.^12^ To accurately evaluate the oncological efficacy of segmental pneumonectomy or subsegmental pneumonectomy, more stringent inclusion criteria needed to be established. This operation meets the following basic requirements: (i) tumor diameter ≤2 cm GGO composition >50%; (ii) multiple peripheral specially located pulmonary nodules in the same lobe; (iii) resection margin larger than tumor diameter; and (iv) intraoperative frozen hilar lymph node pathology.

In this study, pulmonary nodules have a special location, there are two or more small peripheral pulmonary nodules in single lung lobe with segmental resection indication, the position of pulmonary nodules is deep, wedge resection is difficult to ensure enough resection edge, at the same time, pulmonary nodules are far apart, CSS is difficult to remove all pulmonary nodules at the same time. Our team performed a noncombined subsegmental resection of these lung nodules and the subpulmonary segments involved were relatively independent at the same lung lobe, retaining more lung tissue in compliance with the excision indication. Considering the complexity and difficulty of the operation, thorough preoperative planning is needed, which demands higher requirements for the localization of the cutting edge of the focus and the simulation of the intersegmental plane (ISP).^13^, ^14^ Our team used the IQQA‐three‐dimensional image analysis system to obtain three‐dimensional quantitative reconstruction results from the bronchi, pulmonary arteries, and pulmonary veins from CT images, and accurately evaluate the reconstruction by determining whether or not the simulated intersegmental veins followed the simulated ISP.^15^ In practice, the minimum resection range of the target substance is planned according to the spherical boundary established by the IQQA‐3D system with an edge ≥2 cm, and the structure of the target lung segment and the variation of vascular and bronchial branches are accurately explained before the operation, which promotes higher quality preoperative planning and intraoperative navigation.

The method of obtaining and dealing with the intersegmental plane during surgery is one of the key steps in the operation. The reported methods for obtaining the intersegmental plane include selective segmental inflation, systemic injection of indocyanine green, endobronchial dye injection, multidetector CT and virtual‐assisted three‐dimensional lung mapping.^16^, ^17^ However, there are some shortcomings, such as the risk of dye allergy, the risk of air embolism, short dyeing duration, and the need for additional equipment and dark environment.^18^, ^19^ The method of determining the intersegmental plane by the expansion‐collapse line has the advantages of no dye and less equipment, and it can accurately display the inflatable‐venting line and identify the intersegmental vein between the resected segment and the reserved segment. In practice, a favorable and clear intersegmental plane can be obtained by ultrasonic knife separation and stapler separation, which is known as the combined dimensionality reduction method. According to Zheng et al., the combined dimensionality reduction method is helpful to make segmental resection simpler and more accurate, and is expected to become a feasible and effective single‐hole thoracoscopic segmental resection.^20^ According to our experience, the ultrasonic scalpel is used to cut the parenchyma along the expansion‐collapse line, and then the remaining target parenchyma is left through the stapler after “dimensionality reduction”. This avoids the curl and contraction of reserved lung parenchyma caused by direct stapler cutting of three‐dimensional target parenchyma, which is beneficial to the expansion and stretching of lung tissue after operation.

Related studies have shown that for most patients the average decrease in FEV1 after 1–2 segmental resection is unlikely to be of clinical significance, but after 3–5 segmental resection it seems more likely to have adverse clinical effects on overall function and quality of life.^21^ Lung function preservation associated with less resection may be more significant for patients with marginal pulmonary function and those who need additional pneumonectomy to treat metachronous lung cancer in the future. Nomori et al. reported that the median retention rate of pulmonary function after segmental lobectomy was 93.2%, which is significantly higher than that the 85.9% after lobectomy (*p* < 0.001).^22^ According to JCOG0802/WJOG4607L, a randomized controlled trial, the lung function retention rate after segmental lobectomy was significantly higher than after lobectomy (89.6% vs. 86.9%) (*p* < 0.001).^23^ On this basis, simple subsegmental resection or combined subsegmental resection can preserve the pulmonary function of each lobe by avoiding multisegmental resection, especially for tumors in the right upper lobe. In this study, multiple noncombined subsegmental resection of the single lobe was performed, and the adjacent segments were not excised under the safety margin. The postoperative median PPF% of pulmonary function was 91.7%, suggesting that the preservation of pulmonary function can be promoted by noncombined subsegmental resection, so that the postoperative pulmonary function can be maintained in an acceptable range.

To sum up, noncombined subsegmental resection can provide a new and safe method to remove multiple independent subsegments in a single lobe. Although perioperative results are acceptable, further prospective large studies are needed to verify this result.

## FUNDING INFORMATION

Sponsored by key Clinical Specialty Discipline Construction Program of Fujian, P.R.C.
